# Parameterising the effect of human occupancy and kinetic energy on indoor air pollution

**DOI:** 10.1038/s41612-025-01281-9

**Published:** 2026-01-14

**Authors:** Dimitrios Bousiotis, Dylan S. Sanghera, Jenny Carrington, Glyn Hodgkiss, Farzaneh Jajarmi, Khalid Z. Rajab, Francis D. Pope

**Affiliations:** 1 School of Geography, Earth and Environmental Sciences, University of Birmingham, Birmingham, UK; 2 Cundall, Birmingham, UK; 3 School of Electronic Engineering and Computer Science, Queen Mary University of London, London, UK

**Keywords:** Environmental sciences, Environmental social sciences

## Abstract

Indoor air quality (IAQ) is increasingly recognised as one of the most important aspects for public health, workplace safety and productivity. While indoor and outdoor factors both influence indoor pollutant levels, human presence and activity are key drivers of the emission of specific pollutants, including particulate matter (PM), total volatile organic compounds (TVOCs) and carbon dioxide (CO_2_). This study investigates the relationship between occupancy, physical activity measured by kinetic energy (KE), and air pollution concentrations in a real-world office setting, by combining data from air quality and radar motion sensors. Two exemplar office spaces were investigated, comprising an open-office area and a meeting room. PM, in the PM_1_ and PM_2.5_ size fractions, were found to be highly correlated with the outdoor conditions, whereas PM_10_ correlates more closely with indoor occupancy (up to *r* = 0.65). Even higher correlations, up to *r* = 0.74, were found between human activity, quantified as KE, and the PM_10_ concentrations. The TVOCs and CO_2_ showed even stronger correlations with KE (up to *r* = 0.83), suggesting that these metrics can be used as excellent proxies for estimating certain types of indoor air pollution. Notably, the impact of additional occupants varies depending on room characteristics and usage, underscoring the need for contextualised models of IAQ. By quantifying both outdoor infiltration and indoor emissions, this study offers a framework for disentangling pollutant sources and guiding interventions to optimise IAQ. These insights support evidence-based strategies to create healthier and more productive office environments.

## Introduction

The deterioration of air quality (AQ) is one of the most pressing issues affecting both public health, as well as the natural environment^1,2^. While ambient AQ has been extensively monitored and studied, indoor air quality (IAQ) has not historically received the same level of interest. IAQ is, however, of increasing interest^3^, especially since people tend to spend up to 90% of their time in indoor environments; be it their homes, places of work, social and community spaces, or even inside the transport that takes them between the various buildings^4,5^. Poor IAQ has similar negative effects on human health as poor ambient AQ^6^. The World Health Organization (WHO) estimates that out of the 9 million deaths attributed to direct and indirect effects of air pollution, 3.2 million of them are specifically associated with the deterioration of the IAQ^7^.

This value for indoor pollution only considers the deterioration of the IAQ due to substances associated with incomplete combustion of fuel used for cooking. Hence, the total incidence of premature mortality, associated with indoor air pollution from all indoor sources, is almost certainly higher.

The sources of pollution affecting IAQ can be more complicated and harder to decipher than the outdoor ones. Apart from the pollutants generated within the indoor environment, outdoor pollution is also introduced by infiltration through doors and windows, ventilation systems or even cracks in the building shell^8,9^. This makes it harder to understand the role of indoor sources and the range of their effects. Research associated with indoor air pollution is further limited due to the complexity of such campaigns. Until recently, most IAQ-related campaigns were performed using research-grade instruments. Apart from the financial cost that comes with the use of expensive research instruments, the typically large and noisy equipment can cause a great nuisance to the participants’ day-to-day lifestyle and activities. The high pumping rates of research equipment can lead to high air replacement rates within indoor environments, causing more outdoor air infiltration. These downsides to research equipment can lead to indoor campaigns being carried out in highly controlled and in many cases unrealistic setups. Furthermore, the great variability found between the different indoor environments, due to their specifications, conditions and use, resulted in hard to explain^10^ and sometimes contradictory outcomes. However, the emergence of low-cost sensors (LCS) in the last decade can be a possible solution to both problems. Due to their small size, low noise, and ease of setup, they can be used without causing much nuisance to the household, while their low cost both on their price as well as their operation, allows for applications in large numbers, sometimes covering tens or even hundreds of households monitored simultaneously^11,12^. LCS do not come without limitations though, mainly associated with their performance being affected by meteorological conditions^13,14^, while they lack the accuracy and consistency of the more expensive research-grade instruments^15^. However, this is a limitation that does not apply to indoor studies in which the environment is usually conducive to their performance, as the relative humidity, which is the main variable affecting the LCS performance, is in most instances within the optimal performance range of the LCS, thereby facilitating easier calibration. Many indoor studies have highlighted the improved accuracy of the LCS in indoor environments compared to outdoors^16^, making them an ideal option for multi-point indoor campaigns.

Several studies have been undertaken to understand the effects of indoor activities on air quality^17^. In most cases, these are focused on pollutants associated with activities such as cooking, heating, cleaning as they are introducing particulate matter (PM), black carbon and gas pollutants such as NO_2_, CO_2_ and volatile organic compounds (VOCs), which can be harmful to human health^18^. However, one of the most important, yet insufficiently studied, sources of particulate matter within the indoor environment is through the movement of the occupants. PM are usually trapped within the carpeting, furniture, office equipment and the clothes and shoes of the occupants, which are introduced in the indoor environment with resuspension resulting from the movement and activity of the occupants^19–21^. While measurements of PM are very common among indoor studies, the resuspension factor, which mainly affects the PM_10_ concentrations, is often overlooked either due to it being considered unimportant or unavoidable. Nevertheless, PM_10_ which has elevated concentrations in offices and indoor workplaces, can worsen respiratory diseases and asthma and is responsible for about 40% of deaths associated with cardiovascular disease^22^, increasing the hospitalisation cost^23^ and reducing the cognitive function and productivity of workers^24,25^. Many studies have identified the effect of the activity of the occupants on the PM and attempted to decipher it^26^. Bousiotis et al.^27^ not only identified people’s activity as a major source of both PM_2.5_ and PM_10_ in indoor environments but also quantified its effect, which was found to be responsible for up to 60–70% of the total indoor PM_10_ concentrations. A more significant effect of resuspension on coarse particles compared to other indoor sources was also highlighted by Isaxon et al.^28^ and Li et al.^29^. You and Wan^30^ modelled the variable effect of walking speed on PM concentrations in a chamber study, and found a positive correlation between them. These studies considered the effect of human activity on their PM ‘personal cloud’, referring to the emissions from human breathing, clothing, personal care products, moving etc^31^. However, these are not limited to PM, and include CO_2_ from breathing^32^, as well as VOCs derived from personal care products, breathing, saliva and other human excreta^33^.

All these studies identified the activity of the occupants as a potential source of PM in experimental conditions. However, a direct connection between them has not been tested within real-world environments. The present study provides a methodology to connect human activity to air pollution generation within indoor environments. Our novel approach combines air quality and motion sensors to link activity and indoor air quality. In particular, the study uncovers the relationships between the number of occupants and the level of their individual activity, as measured by their kinetic energy (KE), on the variation of the PM, CO_2_ and Total VOCs (TVOCs) concentrations, without the need to consider the specific characteristics of each indoor environment. Furthermore, the infiltration of outdoor PM, mainly affecting the smaller PM_1_ and PM_2.5_, is also considered. By combining the information of the effects of both the infiltrated outdoor pollutants and those generated indoors, the outcomes from such studies provide a more complete image of the factors affecting the indoor air quality, which can be used for its accurate modelling. In this study, we present a simple methodology that can estimate the pollutants’ concentrations within different households and workplaces, providing the information needed for targeted actions to improve the indoor air quality.

## Results

### Particulate matter

The average concentrations of all the PM measured from the outdoor sensor were higher than those found within the two office locations (Table 1). This is as expected, since the office is in the centre of Birmingham, UK, the second-largest city in the UK, and is air-conditioned. The measured indoor to outdoor (I/O) ratios, averaged over all time periods, for the different PM size fractions varied from 0.24 to 0.67, with the value decreasing as the size of particles increased (Table S1). The I/O ratio for PM_2.5_ and PM_10_ in both rooms was significantly lower than those found in the literature for office environments^9,34^. The low I/O ratios likely result from the high concentrations found in the urban outdoor environment as well as the presence of a ventilation system that lowers the PM concentrations within the office areas. The studied open-office area and meeting room presented similar PM concentrations during absent (non-occupied) periods, showing the near uniform effects of the mechanical ventilation system across the wider building space.

**Table 1:** Average and standard deviation of hourly pollutants’ concentrations at the outdoor and indoor sites

Location	Pollutant	ALL	Weekday	Weekend	Present	Absent
Open-office	PM₁ (µg m⁻³)	3.42 ± 2.58	3.32 ± 2.38	3.68 ± 3.02	3.36 ± 2.44	3.49 ± 2.73
	PM_2.5_ (µg m⁻³)	3.69 ± 2.59	3.64 ± 2.40	3.83 ± 3.02	3.76 ± 2.45	3.63 ± 2.73
	PM₁₀ (µg m⁻³)	8.92 ± 8.48	10.9 ± 9.11	3.94 ± 3.00	13.7 ± 9.16	3.75 ± 2.74
	CO₂ (ppm)	522 ± 106	549 ± 113	450 ± 14.8	584 ± 115	454 ± 15.7
	TVOCs (μg m^-3^)	342 ± 274	408 ± 295	170 ± 47.7	495 ± 296	177 ± 94.4
Meeting room	PM₁ (µg m⁻³)	2.94 ± 2.54	2.85 ± 2.36	3.14 ± 2.96	2.71 ± 2.25	3.05 ± 2.67
	PM_2.5_ (µg m⁻³)	3.07 ± 2.56	3.00 ± 2.38	3.24 ± 2.97	2.95 ± 2.28	3.13 ± 2.68
	PM₁₀ (µg m⁻³)	4.63 ± 4.98	5.14 ± 5.48	3.30 ± 2.96	7.40 ± 6.96	3.24 ± 2.70
Outdoor	PM₁ (µg m⁻³)	5.24 ± 3.91	5.17 ± 3.68	5.45 ± 4.46		
	PM_2.5_ (µg m⁻³)	7.97 ± 4.22	7.86 ± 4.18	8.25 ± 4.30		
	PM₁₀ (µg m⁻³)	36.9 ± 120	40.7 ± 141	27.0 ± 19.1		

‘Absent’ refers to the hours when no occupancy was recorded within the motion sensors' detection range

Looking at the weekly variation of the outdoor PM concentrations, on average PM_1_ and PM_2.5_ presented similar concentrations with a negligible increase during the weekend compared to the weekdays. Specifically, for PM_1_ and PM_2.5_, the highest average concentrations were found during Friday and Saturday evening hours, possibly associated with increased leisure activities (restaurants, pubs, etc.) taking place within the city centre at the end of the working week. Contrary, outdoor PM_10_ presented lower concentrations during the weekends.

**Fig. 1:**
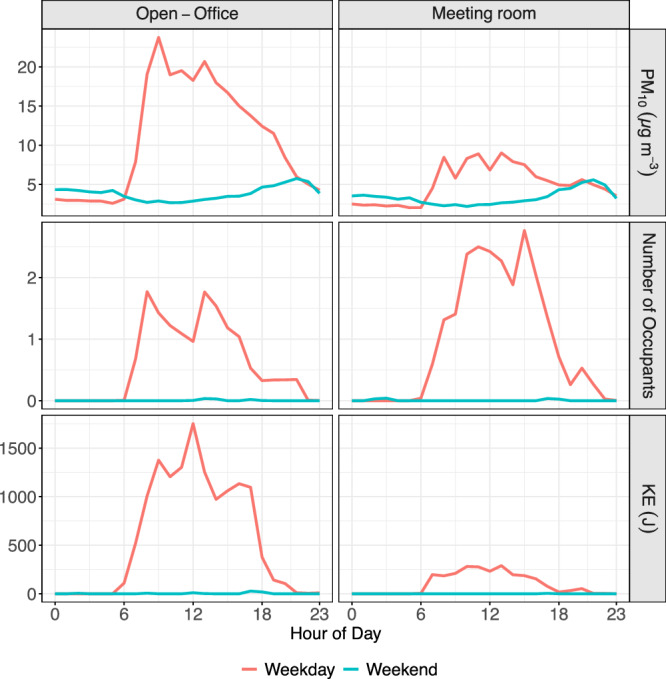
Diurnal variation of PM_10_, occupancy and KE for the open-office area and meeting room.

At the workplace studied, PM_1_ and PM_2.5_ presented a small increase on Friday and Saturday in both rooms, similar to the outdoor trend. Previous studies have shown the importance of outdoor PM infiltrating and shaping the conditions of the indoor environments, especially for smaller particles^35^. Note that, as mentioned earlier, while the I/O ratios are relatively low in this specific environment, the trends for these pollutants in both rooms follow the outdoor ones (Fig. S1) with very high correlations between indoor and outdoor PM_1_ and PM_2.5_ concentrations (Table S2). Conversely, evaluation of indoor PM_10_ trends highlights significant differences between weekdays and weekends (Fig. 1), with the weekday concentrations being up to triple that of the weekends. The presence and activity of people, which is considered as one of the most important sources of PM within indoor environments, appears to be a significant factor in PM concentrations in the studied indoor area, especially within the PM_10_ size fraction. This is further illustrated by the differences between periods when occupants are present or absent in the rooms studied. During the present periods, elevated concentrations of PM_10_ were found compared to periods when the rooms were empty. Typically, PM_10_ concentrations were found to be up to almost four times higher when comparing occupied periods with unoccupied periods. This results in a substantial increase in the I/O ratio of up to 3.8 times, which aligns with evidence of the effect of the indoor sources on the PM_10_ concentrations noted in previous studies. The other pollutants studied, including CO_2_ and TVOCs, also demonstrated elevated concentrations during working days compared to weekends (22% and 140% increase, respectively), specifically during periods where people were present (Fig. S2). Interestingly, PM_1_ concentrations appear to be slightly lower during the periods when the rooms were occupied. Due to the lack of significant indoor sources of PM_1_, such as cooking or heating, this is probably solely associated with the weekly variation of the outdoor PM_1_ concentrations, in which higher PM_1_ concentrations coincide with non-working times for staff of the studied office.

**Fig. 2:**
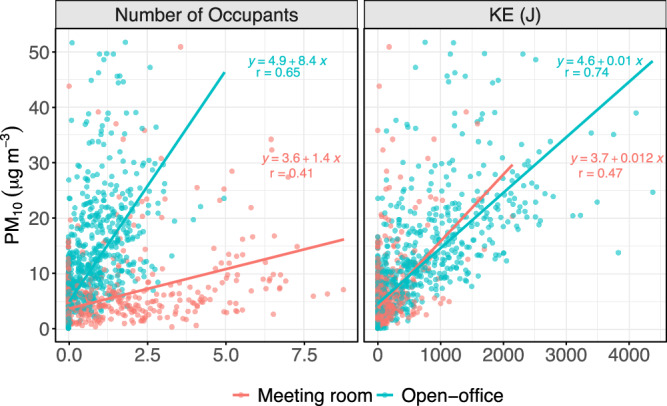
Relationship of occupancy (left) and KE (right) with hourly averaged PM_10_ in each room of the study.

From the previous analysis, it is apparent that the indoor environment is affected by both indoor and outdoor sources of air pollution. Specific pollutants such as PM_10_, CO_2_ and TVOCs present significantly higher concentrations during the times when people are present. To better understand the direct relationships of the pollutants’ concentrations with both the number of occupants and the KE they produce while moving within the indoor environments, we performed a linear regression of these pollutants (the results for PM_1_ and PM_2.5_ are not presented due to low correlations) with the aforementioned variables. It is interesting to note that in Fig. 1, that whilst the meeting room had greater peak occupancy, the walkway (the area directly in front of the sensor monitoring the open-office) had greater peak KE. This is due to the different levels of energy used in the two different environments. Figure 2 presents the relationship of PM_10_ with the occupancy and KE as measured by the motion sensor in the rooms. A relatively high correlation (*r* = 0.65) was found between the number of occupants and the PM_10_ concentrations in the open-office area, a relationship which was not found for the other two size ranges (PM_1_ and PM_2.5_). An even stronger correlation was found between the PM_10_ concentrations and the KE (*r* = 0.74), further confirming the direct effect of people’s movement on PM_10_ emissions.

The analysis in the meeting room did not provide the same level of correlation with the open-office area (Fig. 2). The regressions between the occupancy or KE with the PM_10_ yielded lower correlations, at 0.41 and 0.47 for the occupancy and KE, respectively. This is probably due to the different use of the specific room, as being a meeting room in most cases, it accommodates people who sit down around a table and do not undergo significant movement, reducing their resuspension effect compared to the open-office area, where people move around to talk to colleagues and enter and exit other rooms. Additionally, the windows in the meeting room were sometimes opened, increasing the effect from the infiltration of ambient air. Nevertheless, as indicated by the previous analysis, the presence of people within this room remains the most significant source for PM_10_ as indicated by the comparison between occupied and unoccupied periods.

Using the linear regression between the occupant number and the PM_10_, we can obtain an estimation of the effect of every additional person on the concentrations of the pollutant. In the open-office area, where the correlations were stronger, the additional presence of an occupant on average added about 8.4 μg/m^3^ of PM_10_ to the background concentrations, which were estimated at about 4.9 μg/m^3^. This suggests that it only takes, on average, two or five people to be in the office walkway for the WHO annual or 24-h mean PM_10_ limit recommendations (5 μg/m^3^ or 15 μg/m^3^, respectively) to be surpassed. Whilst workers do not reside in the walkway for either the whole year or whole day, this exemplifies the importance of microenvironments within workplace locations where the working age population spends significant amounts of their lives. The correlation between occupancy and PM_10_ was lower in the meeting room, a smaller effect per occupant on average (~1.4 μg/m^3^) was found at the meeting room due to less activity per occupant in this microenvironment.

Similarly, the effect per KE unit (Joule) was calculated for the two rooms. Thus, about 0.01 μg/m^3^ PM_10_ per Joule of KE produced by the room’s occupants was introduced or resuspended in the open-office area on top of the estimated 4.6 μg/m^3^ background. The similarity of the background estimation between the occupancy and KE parameters, as well as the similarity with the anticipated background (which is expected to be higher during the present periods compared to the absent periods, as continuous resuspension prevents the PM from fully settling), increases the confidence in the outcome of the analysis. Interestingly, an almost identical anticipated effect per Joule of KE was found for the meeting room compared to the open-office area.

A combined dataset of the two rooms was used to estimate the consistency of the occupancy and KE as proxies for the anticipated PM_10_ concentrations between different rooms. Figure S3 shows the linear regression of both occupancy and the KE with the PM_10_ concentrations on the combined dataset. The correlation of the relationship between KE and PM_10_ was higher (*r* = 0.71) than the two individual rooms, indicating that for this specific environment, the movement of the occupants had a similar effect regardless of the room studied.

Unlike for PM_10_, a clear correlation with the occupancy or the KE was not found for PM_1_ and PM_2.5_. This lack of correlation is due to the dominant effect of the outdoor sources on the indoor PM_1_ and PM_2.5_ concentrations. By applying a source apportionment methodology similar to Bousiotis et al.^27^ and isolating the contributions of the indoor sources, we found that indoor sources accounted for <1% and 6% of PM_1_ and PM_2.5_, respectively. Whereas indoor sources accounted for about 40% of the observed PM_10_.

### CO_2_ and TVOCs

**Fig. 3:**
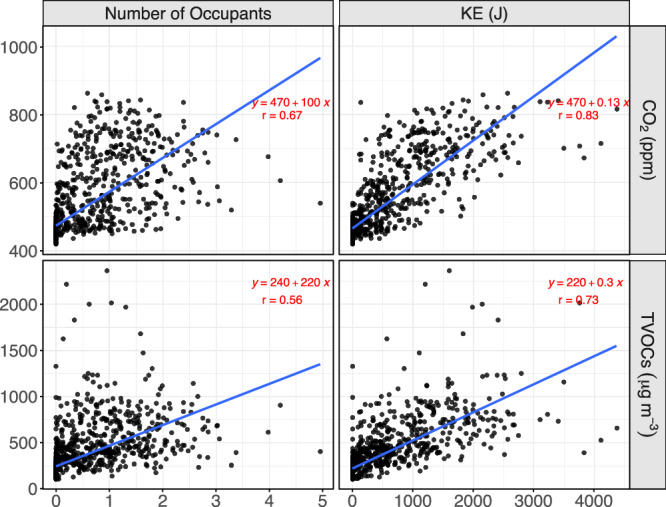
Relationship of occupancy and KE with CO_2_ and TVOCs in the open-office room.

Among the additional pollutants measured in the open-office area, two more were found to correlate strongly with both the occupancy and the KE produced by the occupants. CO_2_, a pollutant which is emitted by human breathing, was found to have even stronger correlations than observed for PM_10_. This is expected as, compared to the PM_10_, the concentrations of CO_2_ are expected to proportionally increase with the number of occupants in a room, as CO_2_ is directly associated with the presence of people and not just their activity. Thus, a Pearson correlation of 0.67 was found between the concentrations of CO_2_ in the room and the number of occupants (Fig. 3). Once again, an even stronger correlation was found with the KE energy (*r* = 0.83), which highlights that not only the number of occupants affects the CO_2_ concentrations, but with increased movement, higher emissions are expected per person as more energy is required with accompanying respiration. Using the linear regression, it was found that from the background concentration of approximately 470 ppm (in both regressions), each occupant added about 100 ppm of CO_2_ for the specific room, while for the KE, it was estimated that each Joule of KE produced by the occupants was associated with an increase of about 0.13 ppm of CO_2_.

Similarly, the variation of the TVOCs measured in the same room presented relatively high correlations with both the number of occupants and the KE produced by them (Fig. 3). As there may be multiple sources of TVOCs in the indoor environment, some of them not associated with the presence of people, these correlations were lower than the ones observed for CO_2_. Nevertheless, correlations of 0.56 and 0.73 were found between the variation of TVOCs with the occupancy and KE, respectively, with the average background estimated to be up to 30% higher than the average measured during the absent period, showing that more diverse sources are associated with this pollutant. Regardless, the estimated added effect of each occupant was calculated at about 220 μg m^−3^, which means that on an average for the specific conditions of the study, it only takes the presence of one person for 1 h to more than double the background concentrations. This explains both the great difference found between the working and non-working hours as well as the great standard deviation on the average concentrations throughout the measuring period. For the relationship with the KE, the estimation of the background was closer to the concentrations during the non-working hours, which may be due to the higher correlations achieved. Finally, the anticipated added TVOCs concentrations per Joule of KE were estimated at about 0.3 μg m^−3^.

## Discussion

While the effect of human activity within the indoor environment on the concentrations of specific pollutants is well established in the literature, there are no clear estimates of the effect of each occupant or their activity on them. This is crucial information when designing the specifications and ventilation solutions for healthy buildings, as well as for the workers’ wellbeing. The assessment of the air quality of indoor environments is challenging due to the differences in specifications, materials and activities that take place within them. While the anticipated effect of the presence of occupants within them was considered in past studies^36^, their level of activity, which plays a crucial role in their contribution to indoor air pollution, was not.

In this study, the correlation between the occupancy, in addition to the occupants’ activity levels, and the air quality of a working environment was studied directly for the first time, through the novel use of both air quality and radar sensors. The results clearly demonstrate that indoor PM_10_, CO_2_ and TVOCs concentrations are mainly affected by the presence and movement of the occupants in the rooms studied. While PM is affected significantly by occupancy and movement, not all size ranges are equally impacted. The smaller-sized PM (PM_1_ and PM_2.5_) follow the outdoor trends with little influence from indoor activities on the overall concentrations.

This study highlights that an approach using the number of occupants alone to estimate indoor air quality is not as effective as considering the kinetic energy produced by the movement of the occupants. The increased accuracy of the estimations achieved with the use of the kinetic energy as a proxy of indoor emitted pollutants improves our understanding of the factors affecting the indoor air quality. Going forward, this new knowledge needs to be coupled with a better understanding of how the size, design and use of different indoor environments will lead to indoor air pollution when workers are placed within them.

This study opens future opportunities for estimating air quality within different indoor environments based upon people’s presence and activity. When coupled with estimations of air pollution infiltration, which is particularly important for PM_1_ and PM_2.5_, the estimation of the effect of the occupants of an indoor environment will provide a more complete assessment of the anticipated indoor air quality conditions, allowing for better strategies for the design and ventilation of the buildings to be designed and adopted. Apart from the pollutants considered here, there are several others which are directly associated with the activity of the occupants of an indoor environment. The approach presented here provides an affordable and easy-to-apply methodology to estimate the factors influencing the concentrations of pollutants in indoor environments, by combining the information of the two sources affecting the indoor air quality, the pollutants infiltrating from outdoors and those generated indoors. By understanding how occupancy and the energy derived from the occupants can affect indoor air quality and considering the infiltration factor, we can better design cleaner and healthier indoor environments. This will improve the quality of life for everyone, and the occupational safety and productivity of the workers in offices and other indoor workspaces.

## Methods

### Study area and material

Projects involving human subjects and activity information in their working environments should ensure the anonymity of the participants unless clearly stated. In this manner, this project received ethical approval from the University of Birmingham Science, Technology, Engineering and Mathematics (STEM) ethics committee, number ERN 3249-Nov2024, and we confirm that the research complied with all relevant ethical regulations, ensuring the anonymity of the participants.

The study took place in two rooms at an office on the fourth floor of a five-storey building at the city centre of Birmingham, UK, over a period of 2.5 months (12/09/2024–22/11/2024). The office last had a major refurbishment in 2014. Three sensors were used to collect air quality measurements along with activity logs provided by motion sensors. Two of the sensors were deployed indoors, in the open-office area and the meeting room, while an outdoor sensor was placed at the top of the building to monitor the ambient conditions (Fig. S4). The open-office area is an open plan area of 10x15m with low-level dividers and desks to accommodate about 20 people, in which the air quality sensor was put in the centre of the room, while the motion sensor was set on the wall facing the main part of the open-office area and the corridor. The second indoor air quality sensor was placed in the meeting room, which is a 4×6 m room with 8 seats for meetings. The motion sensor was placed with the air quality sensor facing the centre of the room, a spot which ensures the monitoring of the occupancy and movement within it. Both rooms had new double-glazed openable windows and are mechanically ventilated (ventilation rate is reported at approximately 1 m^3^/ second per floorplate). A blueprint of the office floor is provided in Fig. S5. The outdoor sensor was set on the roof of the building at a height of about 25 m above ground level.

For the air quality monitoring, the ModulAir by QuantAQ was used. The ModulAir is a modular set of sensors, comprising LCS measuring several air pollutants’ concentrations and meteorological variables. Particulate matter concentrations are measured using a combination of an Alphasense OPC-N3 sensor measuring particulate matter in the range between 0.4 and 40 μm and a Plantower PMS5003 nephelometer, along with LCS for temperature, relative humidity and atmospheric pressure data. As the Alphasense OPC-N3 collects data from several PM size bins, it provides independent PM_2.5_ and PM_10_ concentrations, with a reported detection limit of 0.01 μg m^−3^ and an *R*^2^ accuracy of up to 0.69 and 0.53, respectively, for outdoor measurements (higher for low RH conditions)^37^. The data are collected in a 10-s resolution and then go through the algorithms developed by QuantAQ, calibrating the data and providing a dataset of 1 min resolution which is ready for use without the need for further calibration. Field evaluation showed strong correlations of the calibrated data (*R*^2^ > 0.90) for all PM sizes compared to reference grade instruments^38^.

To detect the occupancy and activity within the rooms, NodeNs Zero motion sensors were deployed along with the QuantAQs. The NodeNs Zero is a motion-detecting 60 GHz radar with 4 cm ranging precision, able to track the presence of up to 10 people within its range of detection. The field of view of the NodeNs Zero is up to 10 m in front of the sensor in a cone with an angle up to 120° (horizontal) and 60° (vertical), depending on the parameters set.

Additionally, in the open-office area, an Airthings Space Pro and an Airthings Wave Plus, monitoring TVOCs using a metal-oxide-based sensor, were used. This sensor detects a broad spectrum of airborne chemicals emitted from sources such as personal care and cleaning products, plants, office equipment etc in the range of 0–10,000 ppb. However, it does not identify specific VOCs but provides an overall indication of TVOCs levels in the environment. It is important to note that the sensor operates on a relative basis, calibrating itself to the cleanest air it encounters over approximately a week. Therefore, regular exposure to fresh air is essential for maintaining accurate readings. The sensors are also able to measure CO_2_ using a Non-Dispersive Infra-Red sensor. The range of CO_2_ measured by the sensor is 400–5000 ppm, with an accuracy of up to ±30 ppm, according to the manufacturer. While these sensors were not calibrated against reference-grade instruments, they have a self-calibration feature using the lowest concentrations monitored as the base for the measurements collected, according to the manufacturer^39^. Since the Airthings sensors were not externally calibrated, the data should be viewed as indicative rather than absolute. The two sensors presented almost identical trends for both pollutants (*r* > 0.97) though they had differences in their readings (a discrepancy of about 10% was continuously observed on both CO_2_ and TVOCs measurements between the two sensors). Thus, the average of their measurements was used for both the TVOCs and CO_2_.

While air quality data were collected continuously for 2.5 months, there are specific periods when motion data were not available due to sensor downtime. These periods were not included in the dataset analysed. Thus, a total of 2527 indoor datapoints of 1-h resolution were used for the analysis, along with an additional 1140 outdoor air quality measurements. For the analysis, the data were averaged to an hourly resolution to reduce the measurement noise and time inconsistencies between the variables considered. PM outliers were removed after the logarithmic normalisation of the dataset according to:

1 $$\mu \pm 2\sigma$$

where *μ* is the mean of the measurements and *σ* is the standard deviation. From the outlier removal process, 3.4% of the measurements were removed. The outliers typically occurred when human activity was very close to the sensor. Hence, the observed high concentration values were not representative of the overall air quality of the room under study at that moment.

### Calculation of the speed and kinetic energy of the occupants

The NodeNs Zero provides the number of occupants within the room for up to 10 people. In the version of the sensor software used in this study, the sensor tracks and provides detailed location information for two occupants. In cases of a greater number of occupants, the sensor tracks and follows the first two occupants in the room, providing a unique ID and 3-dimensional information on their location with a sub-second time resolution, while giving the total number of occupants but no location information for additional people within the same time frame. Once one of the tracked occupants leaves the monitored area, the sensor automatically focuses on another occupant, providing a new ID and tracking their movement instead.

As the sensor provides detailed information on the location of the occupants, their KE as they move within the room can be calculated. It should be noted that since the sensors have a specific monitoring range, the KE calculated using this data does not provide the total KE produced by the occupants in the rooms, but an indication of the activity within them from the area monitored (the calculated KE includes only the part of the occupants’ movement captured by the sensor). The calculation of the KE from the movement of the tracked occupants was based on the KE formula:

2 $${KE}=\frac{1}{2}m\,{v}^{2}$$

where $m$ is the mass of the occupant and $v$ is their speed. For the mass of the occupants an average value of 70 kg was used^40^. The speed was calculated using the distance travelled information deriving from the Pythagorean theorem applied on a 2-dimension plane from the x (horizontal distance from the sensor) and y (vertical distance from the sensor) provided by the sensor, and ranged between almost zero (when people were stationary, as the sensor records micromovements) to 0.52 m/s. As there are no elevated areas within the rooms, the height information, which is also provided from the radar sensors was not considered. The distance travelled between two measurements was calculated as:

3 $$a=\sqrt{{b}^{2}+{c}^{2}}$$

where *a* is the distance travelled, and *b* and *c* are the differences from the starting point to the end point between two measurements for the x and y axes, respectively. By dividing the distance travelled to the time between the measurements we get the speed of the tracked occupants.

While for more than 90% of the measuring period the number of occupants did not exceed this number, cases of more than two occupants were also recorded. In such cases, the average speed of the two tracked occupants in the same room was used for the calculation of the KE of the additional occupants. This may bias the KE generated by the additional occupants, being between near zero and up to four times greater per additional person, depending on their activity (being stationary or walking fast). Nevertheless, this is expected to have a minimal impact on the results due to the rare instances of more than two occupants being present and the use of the average speed for them, reducing the bias from the extremes. Additionally, in the text, the term ‘absent’ refers to periods when no occupants were detected by the motion sensor. However, this does not account only for out of office periods, but also includes periods when people were in the office but not within the detection range of the motion sensors.

## Supplementary information

Supplementary Information

## Data Availability

The datasets generated and analysed during the study have been deposited in the University of Birmingham eData Repository [10.25500/edata.bham.00001330] .
