# Supplementary material for: Parameterising the effect of human occupancy and kinetic energy on indoor air pollution
Source: NPJ Clim Atmos Sci. 2026 Jan 14;9(1):4. doi: 10.1038/s41612-025-01281-9 (PMC12804081; doi:10.1038/s41612-025-01281-9)
Supplement: Supplementary file 1 — Supplementary Information [file 41612_2025_1281_MOESM1_ESM.pdf]

SUPPLEMENTARY INFORMATION

Estimating the effect of human occupancy and activity on indoor air pollution

Dimitrios Bousiotis[1], Dylan S. Sanghera[1], Jenny Carrington[2], Glyn Hodgkiss[2], Farzaneh Jajarmi[2], Khalid Rajab[3], Francis D. Pope[1*]

[1]School of Geography, Earth and Environmental Sciences, University of Birmingham, Birmingham, B15 2TT, UK.
[2]Cundall, Birmingham, B3 2BH, UK.
[3]School of Electronic Engineering and Computer Science, Queen Mary University of London, London, E1 4NS, UK.

*Corresponding Author f.pope@bham.ac.uk

Table S1: Indoor to Outdoor (I/O) ratio of PM_1, $PM_{2.5}$, and PM_{10} in each room, subset by present (occupied) and absent (non-occupied) periods.

**Pollutant**	**Subset**	**Open-office/ Outside**	**Meeting room/ Outside**
PM_1 (µg/m³)	ALL	0.67	0.50
$PM_{2.5}$ (µg/m³)	ALL	0.45	0.36
PM_{10} (µg/m³)	ALL	0.45	0.24
PM_1 (µg/m³)	Present	0.60	0.51
$PM_{2.5}$ (µg/m³)	Present	0.47	0.44
PM_{10} (µg/m³)	Present	0.68	0.41
PM_1 (µg/m³)	Absent	0.74	0.50
$PM_{2.5}$ (µg/m³)	Absent	0.44	0.33
PM_{10} (µg/m³)	Absent	0.18	0.16

Table S2: Pearson correlations between indoor and outdoor PM_1, $PM_{2.5}$, and PM_{10}.

**Pollutant**	**Open-office/ Outside**	**Meeting room/ Outside**
PM_1 (µg/m³)	0.90	0.91
$PM_{2.5}$ (µg/m³)	0.73	0.65
PM_{10} (µg/m³)	0.05	0.00

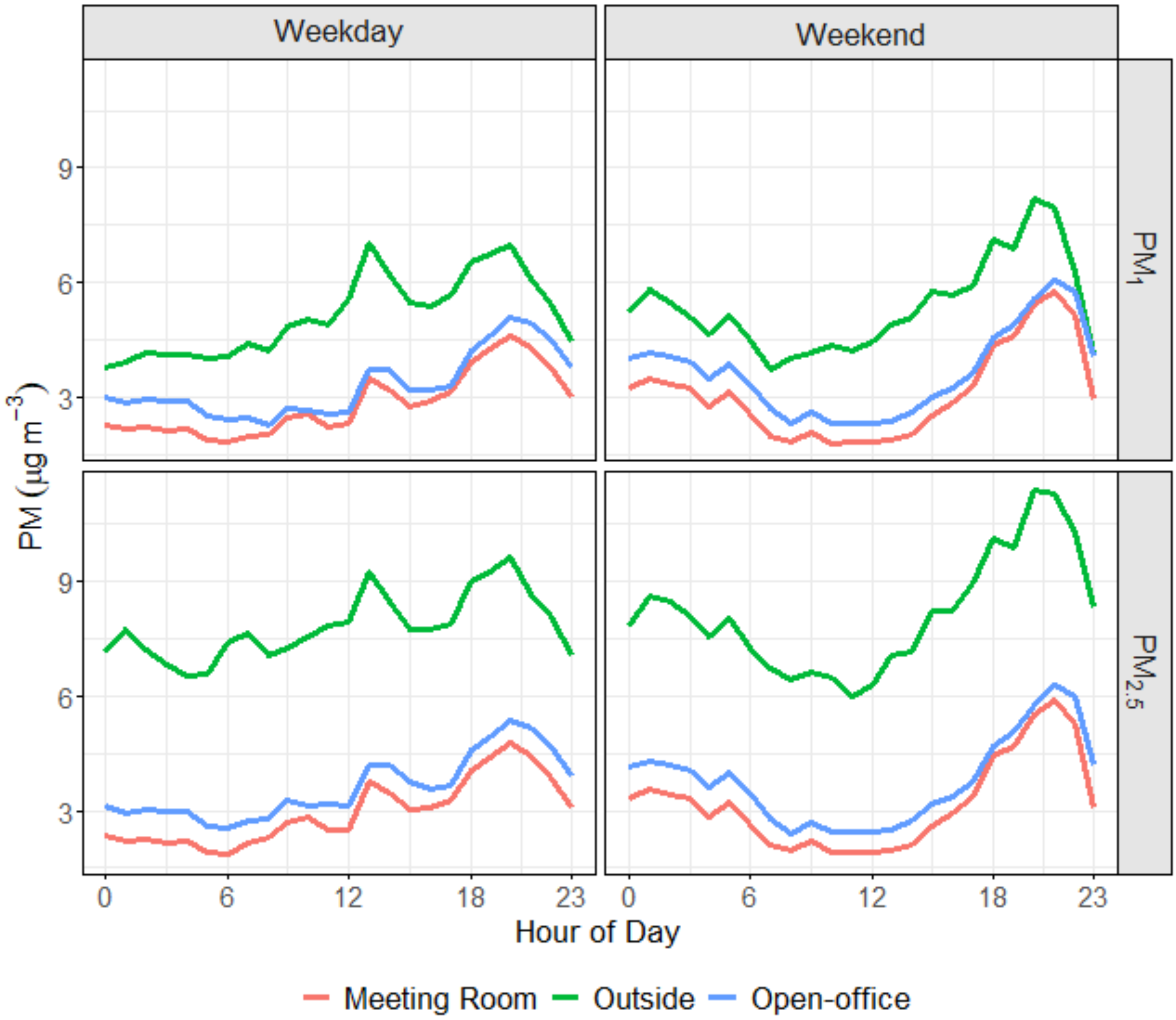

Figure S1: Hourly averaged diurnal variation of weekday and weekend PM_1 and $PM_{2.5}$ in the meeting room, open-office, and outside.

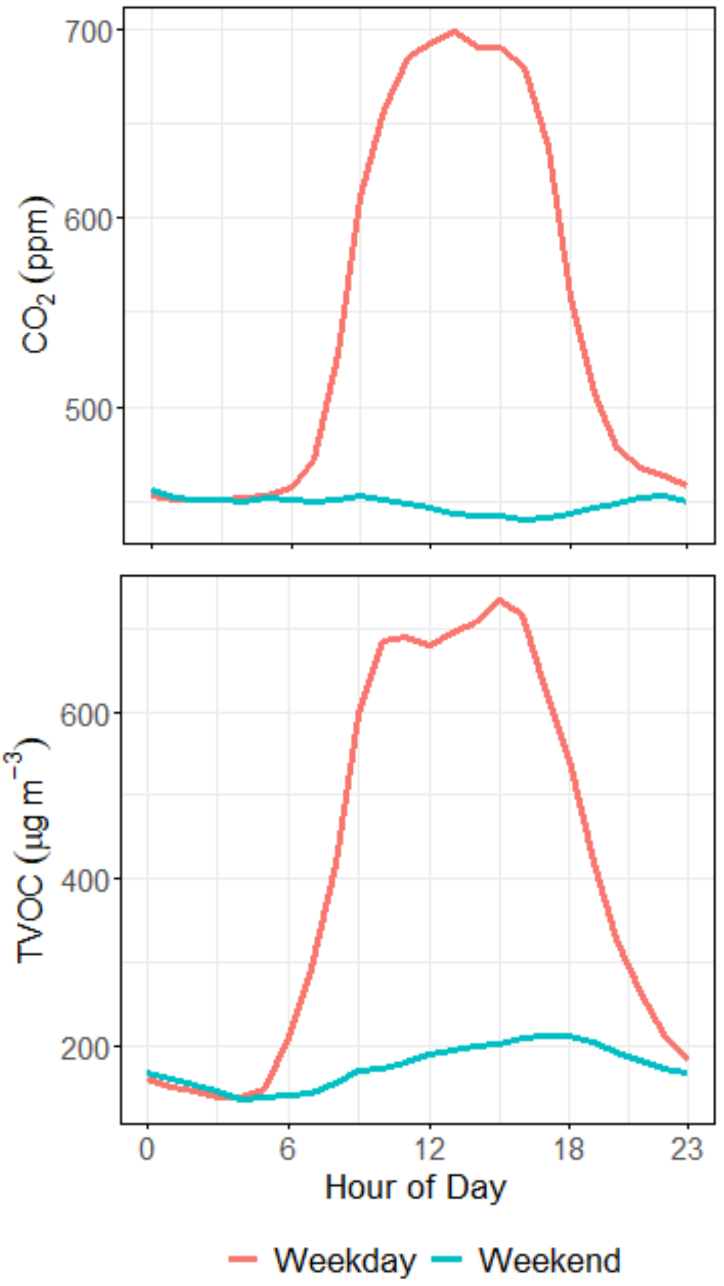

Figure S2: Hourly averaged diurnal variation of CO_2 (top) and TVOCs (bottom) in the open-office for the weekday and weekend.

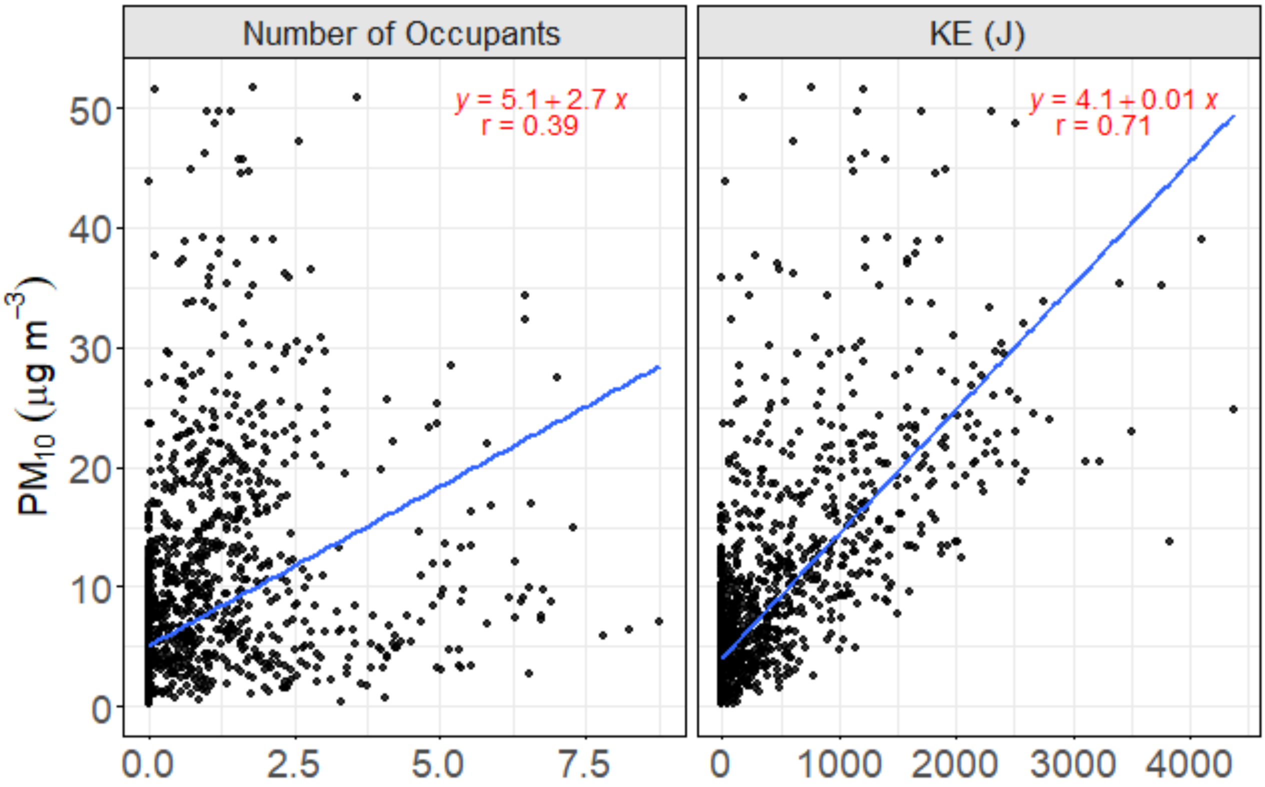

Figure S3: Relationship of occupancy and KE with PM_{10} on the combined hourly averaged dataset, for both rooms.

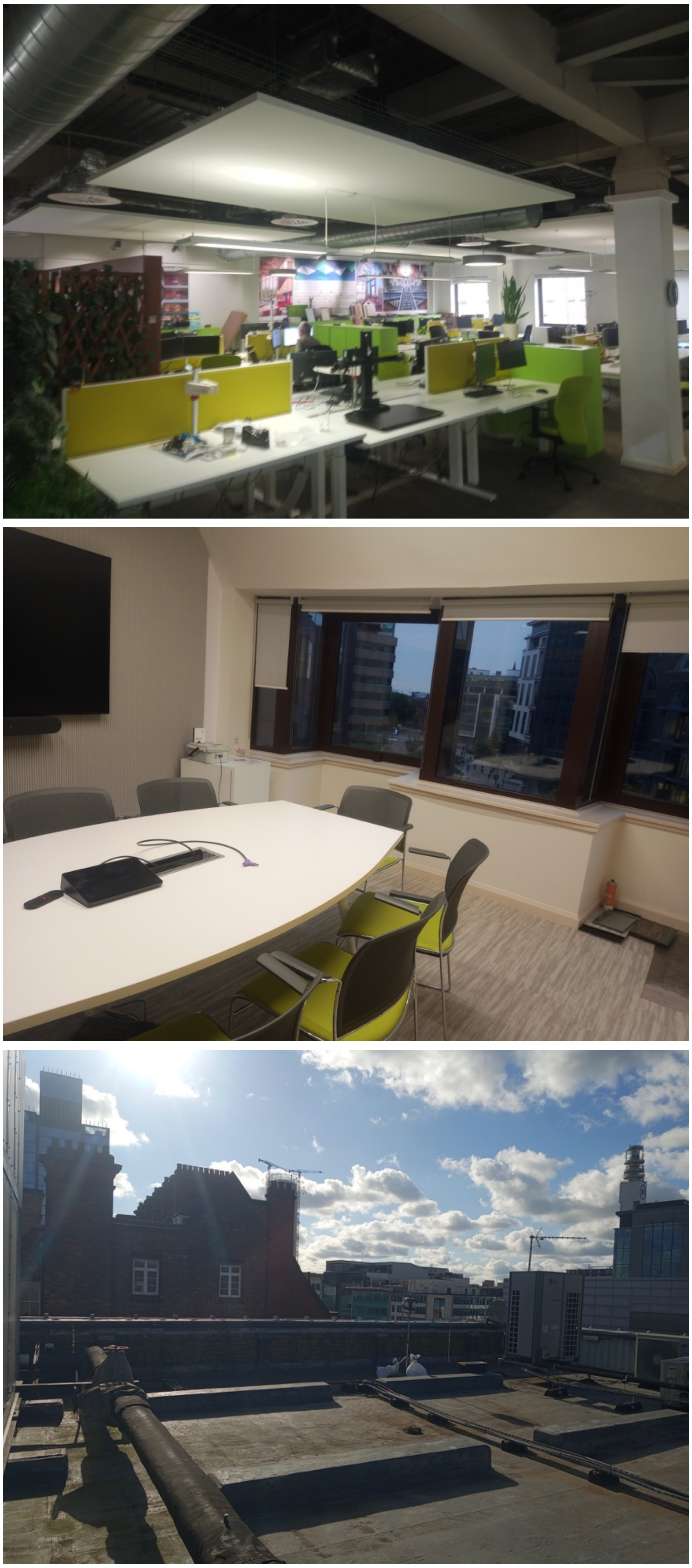

(a)

(b)

(c)

Figure S4: Sensor placement in the (a) office, (b) meeting room and (c) outdoor.

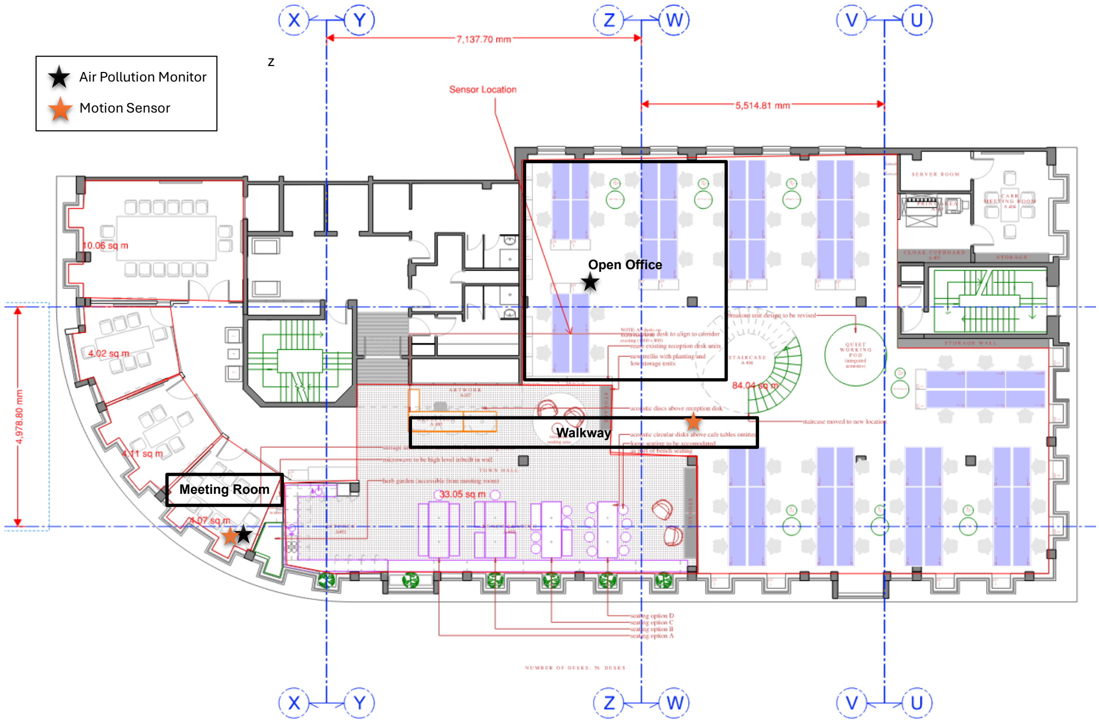

Figure S5: Blueprint of the office floor. Marked are the Open Office, Walkway and Meeting Room areas as well as the location of the sensors.
